# Genetic Patterns of *Myrceugenia correifolia*, a Rare Species of Fog-Dependent Forests of Mediterranean Chile: Is It a Climatic Relict?

**DOI:** 10.3389/fpls.2017.01097

**Published:** 2017-07-06

**Authors:** Fernanda Pérez, Luis F. Hinojosa, Gioconda Peralta, Paz Montenegro, Carla Irarrázabal, Michel Cossio

**Affiliations:** ^1^Departamento de Ecología, Pontificia Universidad Católica de ChileSantiago, Chile; ^2^Institute of Ecology and BiodiversitySantiago, Chile; ^3^Departamento de Ciencias Ecológicas, Universidad de ChileSantiago, Chile

**Keywords:** climatic relict, fog-dependent forest, Mediterranean climate, microsatellites, Pleistocene climatic fluctuations, rare species

## Abstract

Rare species frequently occur in areas with microclimatic conditions that are atypical for their regions, but that were more common in the past, and that probably have operated as climatic refugia for a long time. *Myrceugenia correifolia* is a rare arboreal species that grows in deep canyons and hilltops of the Coast Range of north-central Chile between 30° and 35°S. In the northern edge of its distribution *M. correifolia* grows in small patches of fog-dependent forest surrounding by xeric vegetation. These forest formations are thought to be remnants of an ancient and continuous rainforest that according to some authors became fragmented during aridization of the Neogene (Neogene relict) and to others during warm-dry cycles of the Pleistocene (glacial relicts). Here we asked whether the northernmost populations of *M. correifolia* are Neogene relicts, glacial relicts, or the result of a recent northward colonization. To answer this question we examined genetic diversity and population divergence of *M. correifolia* using microsatellite markers, tested various competing population history scenarios with an approximate Bayesian computation (ABC) method, and complemented these data with ecological niche modeling (ENM). We detected three genetic clusters with a distinctive latitudinal pattern (north, center, and south) and high levels of differentiation (*F*_ST_ = 0.36). Demographic inference supported an admixture event 31 kya between two populations that diverged from an ancient population 139 kya. The admixture time coincides with the beginning of a period of wet conditions in north-central Chile that extended from 33 to 19 kya and was preceded by dry and cold conditions. These results suggest that increased precipitation during glacial periods triggered northward expansion of the range of *M. correifolia*, with subsequent admixture between populations that remained separated during interglacial periods. Accordingly, ENM models showed that suitable habitats for *M. correifolia* in north-central Chile were larger and less fragmented during the Last Glacial Maximum than at present, suggesting that northernmost populations of this species are glacial relicts.

## Introduction

Species with limited geographic distributions are considered more vulnerable to climatic changes and land use conversion than widespread species, and therefore are of central concern in conservation biology (Malcolm et al., 2006; Honnay and Jacquemy, 2007). Rare species frequently occur in areas with microclimatic conditions that are atypical for their regions but were more widespread in the past (Ohlemüller et al., 2008; Harrison and Noss, 2017). These species (or their marginal populations) are thought to be climatic relicts, that is, remnants of past populations that have become fragmented by climate-driven changes (Hampe and Jump, 2011; Woolbright et al., 2014). In arid and semiarid regions, for example, many rare species that currently grow in isolated patches with local humid conditions probably had wider distribution in the past (Herrera, 1992; Thompson, 1999). Fragmentation and contraction of their distribution ranges might be triggered by the increased warming and reduced rainfall that occurred during Neogene (Neogene relicts), or maybe latter, during warm/dry cycles of the Pleistocene (glacial relicts) (Cox and Moore, 2010; Hampe and Jump, 2011; Villagrán et al., 2004).

The Mediterranean region of central Chile, one of the world’s plant diversity hotspots, has high levels of richness and endemism (Villagrán, 1994; Armesto et al., 2007). Many tree species are restricted to the Coast Range, where a chain of remnant forest formations extends as far north as 30°S. At this latitude forest patches are isolated on hilltops surrounded by xeric scrublands and fog interception by plants is the primary source of water (Squeo et al., 2005). Winter rains caused by westerly storm fronts supply increasing humidity southward. The northernmost forest formations, including those of the Fray Jorge–Talinay National Park, have high floristic similarity with temperate forests of southern Chile located 1,000 km to the south that have puzzled biogeographers for a century (Philippi, 1884; Looser, 1935; Muñoz and Pisano, 1947; Schmithüsen, 1956; Croizat, 1962; Troncoso et al., 1980; Villagrán et al., 2004). Some authors have proposed that these forests are relicts of the Neogene (Schmithüsen, 1956; Núñez-Ávila and Armesto, 2006) that became gradually fragmented by the progressive aridization of north and central Chile triggered by the final uplift of the Andes and the establishment of the Humboldt Current. Other authors have proposed that these forests are relicts of range expansions of forest formations in central Chile during glacial periods (glacial relicts) and that became fragmented during interglacial periods (Troncoso et al., 1980; Villagrán et al., 2004). Westerly winds were stronger and displaced equatorward during the Last Glacial Maximum (LGM), intensifying winter rains in Central Chile and causing an expansion of forest formations (Heusser, 1990; Valero-Garcés et al., 2005; Kaiser et al., 2008). Cold/wet glacial conditions shifted to temperatures warmer than today in the early to mid-Holocene, between 14 and 10 kya BP leading to southward contraction of forest formations and expansion of xeric elements (Jenny et al., 2002; Lamy et al., 2004; Maldonado and Villagrán, 2006; Kaiser et al., 2008).

In the fog-dependent forest of north-central Chile arboreal species that are restricted to the Mediterranean region coexist with tree species that reach temperate latitudes. Phylogeographic studies have been conducted in two of them: *Aextoxicon punctatum*, distributed from 30° to 43°S (Núñez-Ávila and Armesto, 2006) and *Drimys winteri*, distributed between 30°S and 55°S (Jara-Arancio et al., 2012). Both species show a strong genetic divergence between populations located north of 32°S and other populations. Although in both studies RAPD markers were used and no time estimation was therefore made, strong divergence has been attributed to the onset of aridity during Neogene (Núñez-Ávila and Armesto, 2006). No phylogeographic studies have been conducted in species of fog-dependent forest with narrow distributions, and it is not known if their northernmost populations may also be considered to be climatic relicts.

*Myrceugenia* is a genus of evergreen trees and shrubs endemic to southern South America with two disjunct dispersal centers, one located in central Chile and the other in south-eastern Brazil (Landrum, 1981). The species that reaches the most northern latitude in Chile is *M. correifolia*. Together with other four species it comprises a clade sister to the remainder of the genus. The crown node of this clade was dated to 24–17 Mya, suggesting that the group evolved before the Neogene aridization (Murillo, 2011; Murillo et al., 2012). *M. correifolia* grows in remnants of forest in the summits and deep valleys of the Coast Range between 30°39′S and 35°05′S and, given its narrow distribution, has been listed as rare (Benoit, 1989) and Endangered (EN, Hechenleitner et al., 2005). Even so, this species forms relatively large populations in the northern edge of its distribution, and together with *A. punctatum* dominates the relict fog-dependent forest formations of Fray Jorge–Talinay and Santa Inés. However, *M. correifolia* is more tolerant to drought and occurs on drier soils (Salgado-Negret et al., 2013).

Here we examined genetic diversity and population divergence of the rare Mediterranean species *M. correifolia* using microsatellite markers. In addition, we used the approximate Bayesian computation (ABC) approach to test different historical scenarios. Finally, we used ecological niche modeling (ENM) to identify the climatic niche of this species under current environmental conditions and to predict its potential distribution during the LGM and mid-Holocene. In this way, we explore the “bioclimatic” connectivity of Coast Range summits during glacial and interglacial periods. We combined this information to assess whether the northernmost populations of *M. correifolia* can be considered: (1) relicts of Neogene that became gradually fragmented by the progressive aridization of north and central Chile; (2) relicts of range expansion during wet conditions of the last glacial period that became fragmented during aridization of Holocene; or (3) the result of a recent northward colonization (this is during Late Holocene). If this species persisted in north-central Chile isolated for a long time, we expect to find strong genetic divergence between northern and southern populations. In contrast, if *M. correifolia* colonized northern areas more recently, we expect to find low levels of populationdivergence, a south-to-north decline in genetic diversity and isolation by distance.

## Materials and Methods

### Sampling

We collected plant material of *M. correifolia* from nine sites located between 30°39′S and 34°41′S in the Coast Range of Chile, covering most of the current distribution range of the species (**Table 1**). A random sample of 15 individuals was collected from each site (when available).

**Table 1:** Location and genetic variation at eight microsatellite loci in 16 sites of *M. correifolia*.

Site	Coordinates (S, W)	*n*	na	*I*	*H*_o_	*H*_e_	*F*_IS_	95% CI
Fray Jorge	30°39′, 71°40′	15	4.71	1.04	0.37	0.52	0.28^∗^	0.17–0.38
Talinay	30°27′, 71°37′	15	4.14	0.93	0.36	0.48	0.25^∗^	0.22–0.29
Co. Santa Inés	32°08′, 71°30′	15	4.86	1.25	0.43	0.64	0.33^∗^	0.20–0.47
Cachagua	33°36′, 71°25′	15	3.14	0.92	0.33	0.53	0.38^∗^	0.30–0.45
Mirasol	33°20′, 71°40′	7	2.43	0.66	0.32	0.40	0.20^∗^	0.08–0.32
Qda. Córdova	33°36′, 71°39′	12	4.00	0.97	0.37	0.53	0.30^∗^	0.20–0.36
Tanumé	34°09′, 71°59′	15	3.14	0.75	0.54	0.43	-0.26	-0.30 to -0.15
Cahuil	34°35′, 72°00′	15	2.57	0.63	0.35	0.38	0.08	-0.09 to 0.18
LoValdivia	34°41′, 72°00′	15	3.00	0.70	0.29	0.40	0.28	0.23–0.38

*Shown for each population are: sample size (*n*), mean number of alleles per locus (na), Shannon’s information index (*I*), observed (*H*_*o*_) and expected (*H*_*e*_) heterozygosity, inbreeding coefficient (*F*_*IS*_), and 95% bootstrap confidence intervals of *F*_*IS*_. Asterisk indicates *F*_*IS*_ values significantly higher than 0.*

### DNA Extraction and Amplification

Genomic DNA was extracted from each sample using the DNEasy Plant mini kit (Qiagen, Hilden, Germany). A total of 124 individuals were genotyped for eight microsatellite loci (P3, P4, P5, P6, P9, P11, P15, P17), using the primers and protocol described by Pérez et al. (2014). PCRs were carried out in 10 μl reaction volumes containing 5 ng of template DNA, 1.6 pmol reverse primer, 0.8 pmol M13-tailed forward primer (M13 forward sequence and microsatellite forward primer), 1.6 pmol fluorescently labeled (6-FAM, 1-vic or NED) M13 universal primer, Taq DNA polymerase (GoTaq, Promega), 5 μL 2× GoTaq Master Mix (supplied with the enzyme). Cycling conditions consisted of an initial denaturing step of 5 min at 95°C, followed by 30 cycles of 30 s at 95°C, 45 s at 52°C, 45 s at 72°C, and a final elongation step at 72°C for 10 min. For genotyping, 1 μl of the PCR product was added to 22 μl formamide and 0.5 μl LIZ-400 size standards. The mixture was run on the ABI PRISM 310 (Applied Biosystems), and analyzed using Peak Scanner^TM^ Software version 1.0 (Applied Biosystems).

### Genetic Diversity

For each site we estimated the mean number of alleles (*N*), number of rare alleles (AR), expected heterozygosity (*H*_e_), observed heterozygosity (*H*_o_), and deviations from Hardy–Weinberg using GenAlEx version 6 (Peakall and Smouse, 2006). We also estimated the inbreeding coefficient (*F*_IS_) and its 95% confidence interval (CI) using the R package diveRsity (Keenan et al., 2013). CIs were obtained using 999 bootstrap replicates. To examine possible founder effects and explore the process of postglacial expansion, we tested the effect of latitude on within-population genetic diversity (locality of Mirasol with a small sampling size was excluded from analysis; **Table 1**). Analyses were performed in R (R Core Team, 2014).

### Population Divergence

Genetic differentiation within and among populations was measured by analysis of molecular variance (AMOVA) (Excoffier et al., 1992) using GenAlEx version 6 (Peakall and Smouse, 2006). Population genetic structure was also examined using STRUCTURE version 2.1 (Pritchard et al., 2000). This analysis uses a Bayesian approach to identify the number of genetic clusters (*K*) and assign probabilistically each individual to these clusters without *a priori* knowledge of putative populations. Ten independent runs were carried out for *K* ranging from 1 to 9 using a Markov Chain Monte Carlo run length of 500,000, a burn-in of 50,000 and an admixture ancestry model assuming correlated allele frequencies. To determinate the most likely number of clusters (*K*) we used the rate of change in Ln P(D) (the log probability of data) between successive *K* values as suggested by Evano et al. (2005). To quantify the degree of differentiation between these clusters we performed a hierarchical AMOVA in GenAlEx. In order to identify patterns of isolation by distance (IBD) we estimated pairwise *F*_ST_ values for all pair of sites in GenAlEx, and then performed a Mantel test with geographic distances. In order to discount those clusters obtained by STRUCTURE resulted from simple IBD, we conducted a partial Mantel test between genetic distances and clusters using geographic distances as covariate (Meirmans, 2012). Given that clustering and demographic analyses can be sensitive to linkage disequilibrium (LD; Putman and Carbone, 2014), we examined the level of LD between each pair of loci in Genepop version 4.2 (Raymond and Rousset, 1995; Rousset, 2008). We performed probability tests in each population and across all populations using default Markov chain parameters. No statistically significant LD was detected; either when each population was tested separately (all *p*-values >0.09) and when all populations were pooled (all *p*-values >0.10).

### Demographic History

**FIGURE 1:**
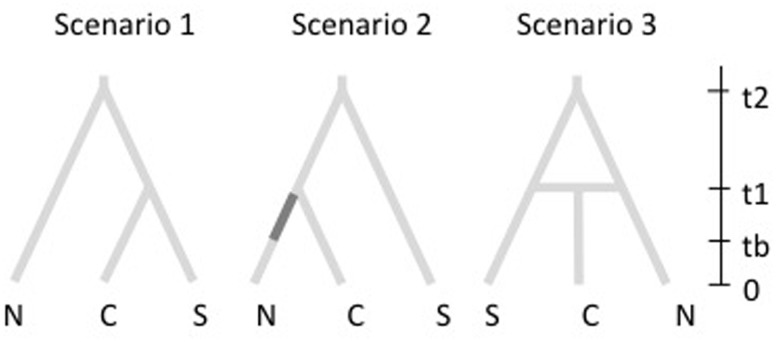
Scenarios explored in DIYABC. In the first, northernmost populations diverged earlier than central and southern populations. The second scenario corresponds to a sequential colonization northward, and includes an initial bottleneck (indicated by the darker section in the N branch of second tree). The third scenario includes an admixture event that resulted from range expansion of two separated populations.

To reconstruct the history of population divergence of *M. correifolia* we used coalescent-based ABC implemented in DIYABC v2.0 (Cornuet et al., 2014). This analysis allows for the comparison of different historical scenarios regarding the order of population divergence, admixture and changes in population size. Based on results of Structure analysis, we defined three populations and constructed three scenarios. The first was designed to fit an ancient divergence of northernmost populations, followed by a posterior divergence of central and southern populations, and is based on the hypothesis that the northernmost populations of *M. correifolia* are Neogene relicts. The second scenario reflects a sequential colonization northward. We assumed that the founder populations were small, and therefore we included an initial bottleneck (**Figure 1**). The third scenario includes an admixture event that resulted from range expansion of two populations that diverged from an ancient population. This scenario is based on the hypothesis that increased precipitation during glacial periods triggered range expansion of *M. correifolia*, with subsequent admixture between populations that remained separated during the last interglacial period (LIG). We set uniform priors for all demographic parameters. The microsatellite loci were divided into two groups: (1) all dinucleotide repeat loci and (2) the single trinucleotide repeat locus. We ran 4 × 10^6^ simulated datasets for each scenario to estimate posterior probabilities using logistic regression. We selected the scenario with the highest posterior probability and estimated the values of the associated demographic parameters.

### Climatic Niche

We estimated the realized climatic niche of *M. correifolia* from a total of 18 unique-georeferenced species records. We obtained the occurrences from national herbaria: Universidad de Concepción (CONC); Museo Nacional de Historia Natural (SGO), online database Global Biodiversity Information Facility (GBIF); Cooperative Taxonomic Resource for American Myrtaceae (CoTram) and our own field work. We modeled the climatic niche employing the maximum entropy method (Phillips et al., 2006, 2017) using 19-bioclimatic variables from WorldClim v1.4 data set (Hijmans et al., 2005). A total of 20,000 background points were randomly chosen within the area comprised between 25° and 38°S in Chile, which includes the Mediterranean climate. Occurrence data were partitioned 100 times into training and test data (75 and 25%, respectively) for model evaluation using the operating characteristic curve (AUC). Resolutions of environmental variables were 2.5 arc-minutes (approximately 4.5 km^2^). For the LGM and mid-Holocene model projections we used the pre-industrial calibrated Model for Interdisciplinary Research on Climate (MIROC-ESM; Watanabe et al., 2011), downscaled at 2.5 arc-minutes (Hijmans et al., 2005).

## Results

### Genetic Diversity and Population Divergence

**FIGURE 2:**
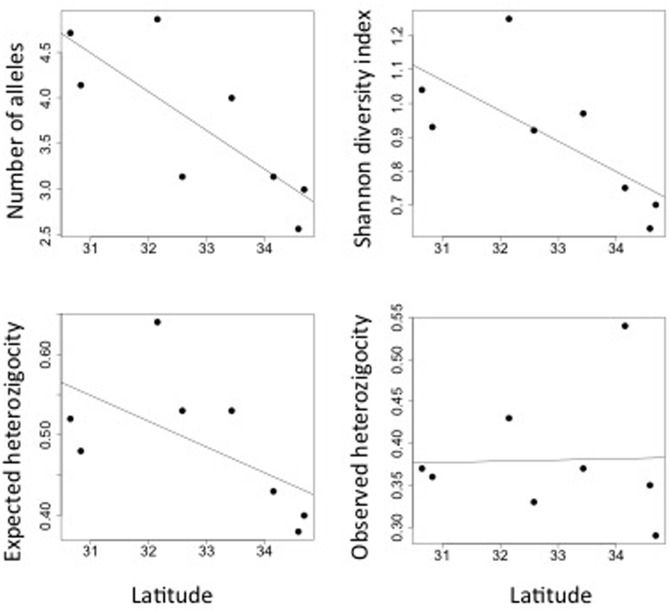
Latitudinal variation in genetic diversity of *M. correifolia* along the Chilean Coast Range.

**FIGURE 3:**
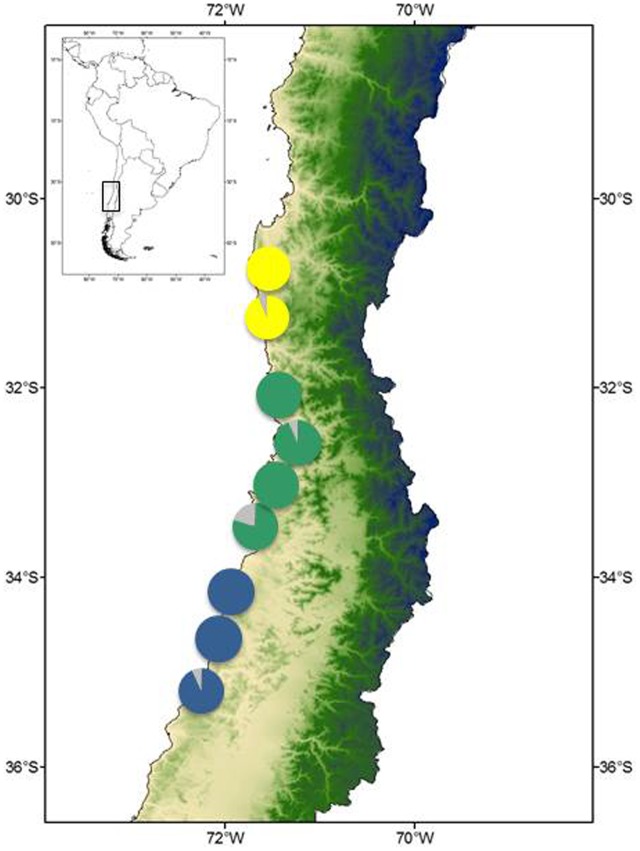
Population structure inferred from STRUCTURE. Colored pie charts show the proportional membership in each of the *K* = 3 clusters identified for *M. correifolia*.

**FIGURE 4:**
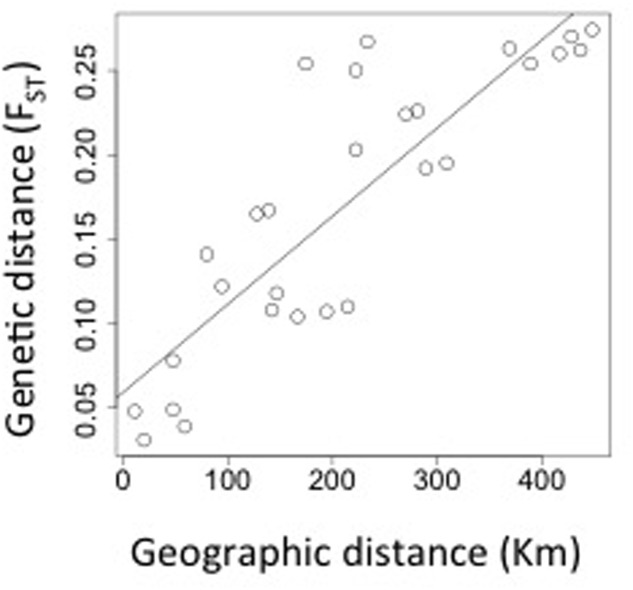
Pairwise genetic differentiation among populations (*F*_ST_) according to geographic distance.

*Myrceugenia correifolia* showed a decreasing trend in genetic diversity southward (**Table 1** and **Figure 2**). Regression analyses revealed a significant effect of latitude on allelic richness (*F*_1,6_ = 11.7, *p* = 0.01), Shannon genetic diversity index (*F*_1,6_ = 32.7, *p* = 0.001), *H*_e_ (*F*_1,6_ = 11.72, *p* = 0.01), but not on *H*_o_ (*F*_1,6_ = 0.15, *p* = 0.70). Three genetic clusters were detected in *M. correifolia* by Structure analysis with a distinctive latitudinal pattern: (1) north, with the sites Fray Jorge and Talinay; (2) center, with the sites Santa Inés, Zapallar, and Qda. Córdova; and (3) south, with the remaining sites (**Figure 3**). Most individuals (94%) were assigned to a single genetic cluster (80 of inferred ancestry) and AMOVA indicated high differentiation among regions, with 36% of the variation explained by differences among them and only 8% by differences among populations. Divergence between north and central clusters (*F*_ST_ = 0.22) was weaker than the barrier between central and south clusters (*F*_ST_ = 0.31). The Mantel test between pairwise *F*_ST_ values and geographic distances revealed a strong and significant relationship between geographic and genetic distance (*F*_ST_; *r* = 0.86, *p* = 0.001), suggesting a significant IBD pattern (**Figure 4**). A significant association between genetic distances and clusters (partial Mantel test: *r* = 0.72, *p* = 0.003) was also detected when geographic distance was included as a covariate, indicating the presence of additional barriers to gene flow.

### Coalescence Analyses

DIYABC analyses indicated that the most likely scenario is scenario 3, which describes an admixture event that resulted from range expansion of two populations that diverged from an ancient population (**Figure 1**). This scenario had a probability of 0.53 with a 95% CI ranging between 0.51 and 0.56 that did not overlap with that of scenario 2 (probability = 0.34; 95% CI = 0.32–0.38) and scenario 1 (probability = 0.10; 95% CI = 0.08–0.13). According to scenario 3, admixture occurred 6,330 generations ago (90% CI = 1,380–12,100) from two populations that diverged from an ancient population 27,800 (90% CI = 1,380–2,100) generations ago. The rate of admixture was higher between north and central populations (*r* = 0.64) than between the central and southern groups (1 -*r* = 0.36). The effective population size was greater in the central group (*N*_e_ = 21,200; 90% CI = 8,000–29,000) than in northern (*N*_e_ = 12,800; 90% CI = 4,460–24,300) and southern groups (*N*_e_ = 4,360; 90% CI = 1,510–10,200).

### Niche Modeling

**FIGURE 5:**
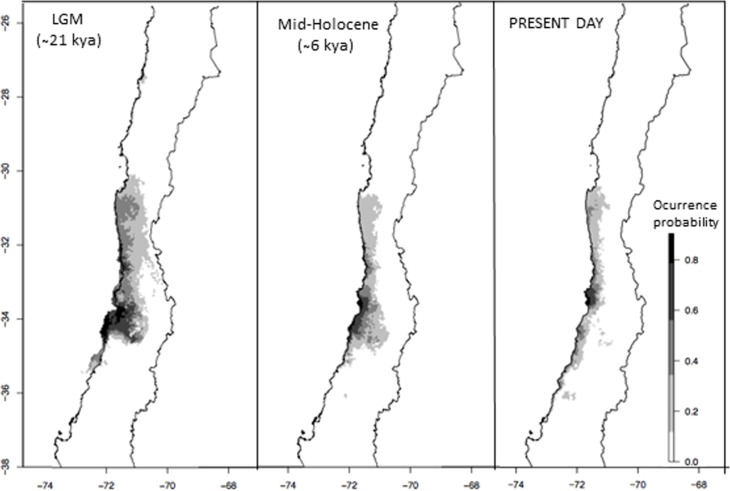
Geographic distribution of *M. correifolia* according to MPI ESM-P model consensus maps for the Last Glacial Maximum, mid-Holocene and the present.

The ENM model for *M. correifolia* showed high power of discrimination between presences and background, with AUC values of 0.99. Under present conditions we detected an area of high suitability at 33°S and several areas of moderate suitability across the known distribution of *M. correifolia* (**Figure 5**). Paleoclimatic models suggested that areas of high and moderate suitability were larger and less fragmented during the LGM than at present. The potential distribution of *M. correifolia* during mid-Holocene was similar to the present one, but with a larger area of high suitability, extending between 33° and 34°S (**Figure 5**).

## Discussion

Fog-dependent forests that today grow isolated on tops of the Coast Range of Central Chile are thought to be remnants of an ancient and continuous rainforest that according to some authors became fragmented during aridization of the Plio-Pleistocene transition (Schmithüsen, 1956; Núñez-Ávila and Armesto, 2006), and to others during warm-dry cycles of the late (Looser, 1935; Skottsberg, 1948) or early Pleistocene (Muñoz and Pisano, 1947; Troncoso et al., 1980; Villagrán et al., 2004). Our microsatellite data for *M. correifolia*, one of the dominant trees of these fog-dependent forests, suggest that divergence of its northernmost populations located in the emblematic Fray Jorge–Talinay National Park was not as ancient as previously proposed.

Our results indicated that increased precipitation during glacial periods triggered range expansion of *M. correifolia*, with subsequent admixture between populations that remained separated during interglacial periods. According to DIYABC analysis, admixture occurred 6,330 generations ago between two populations that diverged from an ancient population 27,800 generations ago. The generation time of natural populations of *M. correifolia* remains unknown, but there are data for individuals grown in the National Botanical Garden of Chile (Mauricio Cisternas, personal communication), indicating that they can reach reproductive maturity at 5 years. Assuming a generation time of 5 years, admixture would have occurred 31 kya and ancient divergence at 139 kya. Admixture time coincides with the beginning of a period of wet conditions in north-central Chile that extended from 33 to 19 kya and that was preceded by dry and cold conditions (Kaiser et al., 2008). Enhanced humidity during this period is supported by marine and terrestrial proxies showing expansion of typical evergreen arboreal taxa at 34°S (Heusser, 1990; Valero-Garcés et al., 2005) and increase in abundance of humid-adapted C3 vegetation at 30°S (Hebbeln et al., 2002; Kaiser et al., 2008). Increased winter precipitation was also revealed by an extensive glacial advance dated at 32 kya recorded in the Cordon de Doña Rosa at 30.7°S, where conditions during the LGM were too arid to allow significant glacial events (Zech et al., 2007).

Divergence time (i.e., 139 kya) is somewhat earlier than the beginning of the LIG. This period, extending between 115 and 130 kya, was characterized by global warming, sea level rise and increased summertime-insolation (Cape-Last Interglacial Project Members, 2006; Dutton and Lambeck, 2012). The increase in temperature was more pronounced in the Artic and Antarctica, where surface temperatures were up to 8°C (Neem Community Members, 2013) and 5°C (Epica Community Members, 2006) warmer than today. Marine and terrestrial proxies also suggest warmer conditions in mid- and high-latitudes of the Northern and Southern Hemispheres (Turney and Jones, 2010). In the case of central Chile pollen data suggest that conditions during LIG were more arid and possibly warmer than the current interglacial (Heusser et al., 2006). To predict the potential distribution of *M. correifolia* during LIG, we performed ENM using the general circulation model built by Otto-Bliesner et al. (2006). Unexpectedly, we found that the potential latitudinal range of *M. correifolia* during LIG do not differ substantially from its current range (Supplementary Figure 1). However, this result is in line with several studies showing that general circulation models tend to underestimate the magnitude of LIG warming respect to proxy reconstructions (Lunt et al., 2013; Otto-Bliesner et al., 2013; Bakker et al., 2014). For the Southern Hemisphere, general circulation models actually give equivocal signals or near zero trends (Lunt et al., 2013; Bakker et al., 2014).

We propose that range expansion during the last glacial period was followed by range fragmentation during the early to mid-Holocene, when conditions were warmer than today (Maldonado and Villagrán, 2006; Kaiser et al., 2008), and according to ENM results, areas of high climatic suitability contracted southward (**Figure 5**). We detected a genetic barrier at 31.5°S separating Fray Jorge and Talinay from the remaining sites of *M. correifolia*. A similar disjunction has been reported for *D. winteri* (Jara-Arancio et al., 2012) and *A. punctatum* (Núñez-Ávila and Armesto, 2006), but in the last case the barrier is slightly farther south and separates Fray Jorge and Santa Inés from the remaining sites. Although in both studies RAPD markers were used and no time estimation was therefore made, Núñez-Ávila and Armesto (2006) attributed this divergence to the onset of aridity during Neogene. Unlike *M. correifolia*, these species have a widespread distribution and are able to grow in temperate forests. Both species are less tolerant to drought than *M. correifolia* (Salgado-Negret et al., 2013) and might have colonized Fray Jorge and Talinay in a different period. Further studies with similar molecular markers are needed to assess whether temperate and rare species that today coexist in the emblematic forest of Fray Jorge and Talinay diverged at different times.

Structure analysis also revealed a second barrier at 33.5°S, separating the southern group from the other populations of *M. correifolia*. This group had a smaller effective population size (*N*_e_ = 4,360) than the central (*N*_e_ = 21,200) and northern groups (*N*_e_ = 12,800). Reduction in genetic diversity southward has been documented in species from north-central (Ossa et al., 2013) and southern Chile (Núñez-Ávila and Armesto, 2006; Segovia et al., 2012), and has been attributed to signals of postglacial expansion. This scenario is supported by ENMs results, showing a reduction in probability occurrence south of 34°S during LGM (**Figure 5**).

## Author Contributions

FP conceived and designed the study, analyzed and interpreted data, and wrote the paper. CI, MC, and GP obtained and analyzed microsatellite data. PM performed niche modeling analysis. LH designed the study and interpreted data.

## Conflict of Interest Statement

The authors declare that the research was conducted in the absence of any commercial or financial relationships that could be construed as a potential conflict of interest.
