# Supplementary material for: Genetic Patterns of Myrceugenia correifolia, a Rare Species of Fog-Dependent Forests of Mediterranean Chile: Is It a Climatic Relict?
Source: Front Plant Sci. 2017 Jul 6;8:1097. doi: 10.3389/fpls.2017.01097 (PMC5498513; doi:10.3389/fpls.2017.01097)
Supplement: Supplementary file 1 [file Image_1.pdf]

Supplementary Material

GENETIC PATTERNS OF MYRCEUGENIA CORREIFOLIA, A RARE SPECIES OF FOGDEPENDENT FORESTS OF MEDITERRANEAN CHILE: IS IT A CLIMATIC RELICT?

Fernanda Pérez, L. Felipe Hinojosa, Gioconda Peralta, Paz Montenegro, Carla Irarrázaval, Michel Cossio

* **Correspondence**: Fernanda Pérez: mperezt@bio.puc.cl

Supplementary Figures

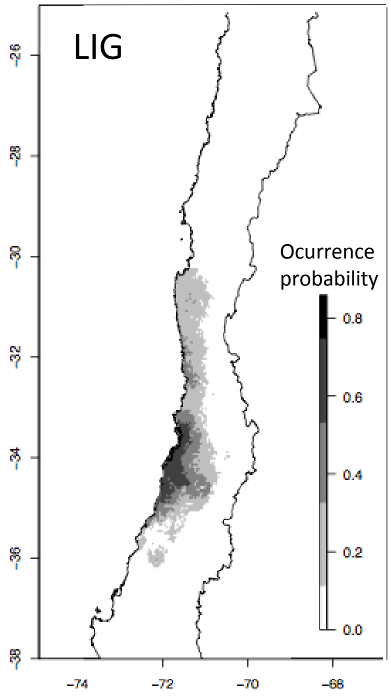

Supplementary Figure 1. Potential distribution of *M. correifola* during the Last Interglacial according to the model generated by Otto-Bliesner et al. (2006).
